# Identification of Radiotherapy-Associated Genes in Lung Adenocarcinoma by an Integrated Bioinformatics Analysis Approach

**DOI:** 10.3389/fmolb.2021.624575

**Published:** 2021-06-15

**Authors:** Junhao Wang, Qizheng Han, Huizi Liu, Haihua Luo, Lei Li, Aihua Liu, Yong Jiang

**Affiliations:** ^1^ State Key Laboratory of Organ Failure Research, School of Basic Medical Sciences, Southern Medical University, Guangzhou, China; ^2^ Department of Respiratory and Critical Care Medicine, Nanfang Hospital, Southern Medical University, Guangzhou, China

**Keywords:** lung adenocarcinoma, radioresistance, immune cell infiltration, bioinformatics, drug candidate

## Abstract

Radiotherapy (RT) plays an important role in the prognosis of lung adenocarcinoma (LUAD) patients, but the radioresistance (RR) of LUAD is still a challenge that needs to be overcome. The current study aimed to investigate LUAD patients with RR to illuminate the underlying mechanisms. We utilized gene set variation analysis (GSVA) and The Cancer Immunome Atlas (TCIA) database to characterize the differences in biological functions and neoantigen-coding genes between RR and radiosensitive (RS) patients. Weighted Gene co-expression network analysis (WGCNA) was used to explore the relationship between RT-related traits and hub genes in two modules, i.e., RR and RS; two representative hub genes for RR (MZB1 and DERL3) and two for RS (IFI35 and PSMD3) were found to be related to different RT-related traits. Further analysis of the hub genes with the Lung Cancer Explorer (LCE), PanglaoDB and GSVA resources revealed the differences in gene expression levels, cell types and potential functions. On this basis, the Tumor and Immune System Interaction Database (TISIDB) was used to identify the potential association between RR genes and B cell infiltration. Finally, we used the Computational Analysis of Resistance (CARE) database to identify specific gene-associated drugs for RR patients and found that GSK525762A and nilotinib might be promising candidates for RR treatment. Taken together, these results demonstrate that B cells in TME may have a significant impact on the RT and that these two drug candidates, GSK525762A and nilotinib, might be helpful for the treatment of RR patients.

## Introduction

Lung cancer is one of the deadliest malignancies in the world. Eighty-five percent of lung cancer patients suffer from non-small-cell lung cancer (NSCLC) and are in the advanced stage, requiring more aggressive treatment options (Price, 2012; Kumarakulasinghe et al., 2015). Histopathologically, NSCLC can be divided into lung adenocarcinoma (LUAD) and lung squamous cell carcinoma (LUSC). LUAD is the most common subtype, accounting for more than 50% of all NSCLC cases (Kumarakulasinghe et al., 2015; Faruki et al., 2017). The interaction between immune cells and the tumor microenvironment (TME) is highly dynamic in different steps of cancer progression (Xiao et al., 2019; Deberardinis, 2020; Menzel and Black, 2020). Although the microenvironment of normal tissue can inhibit tumor growth, changes induced by tumor cells influence this microenvironment to promote tumor progression and metastasis (Wallace et al., 2013). In addition, numerous studies have shown that the tumor-infiltrating immune cells play a pivotal role in the TME (Chen et al., 2020).

Radiotherapy (RT) could not only reduce tumor burden to the minimum but also trigger the anti-tumor immunity and reprogram the TME (Liu et al., 2020). However, Radioresistance (RR) limits the efficacy of RT through TME alteration during fractionated RT (Gu et al., 2019). Several studies have shown that interactions between multiple cells in the TME are the main cause of treatment tolerance to RT (Yang et al., 2019). Nevertheless, the molecular mechanism of radioresistant LUAD is not yet well understood.

Over the past 25 years, high-throughput sequencing technologies have been used to explore the mechanism of tumorigenesis (Cancer Genome Atlas Research Network, 2014; Girard et al., 2016). On this basis, a series of immune-related prediction algorithms have been developed (Rigden and Fernandez, 2020), which provide an excellent chance to explore the TME of various tumors. To explore the mechanism of RT in LUAD and provide helpful advice for treatment, in this study, we performed integrated bioinformatic analysis of LUAD and identified functional differences among RT-treated LUAD patients after gene set variation analysis (GSVA) with The Cancer Genome Atlas (TCGA) data.

The Cancer Immunome Atlas (TCIA) was used to analyze tumor neoantigen-coding genes associated with TME differences among individual RT patients. Furthermore, we performed weighted gene co-expression network analysis (WGCNA) to identify the key modules related to clinical characteristics and ClueGO to assess the potential function of these modules. Four hub genes, including RR module genes (MZB1 and DERL3) and radiosensitive (RS) module genes (PSMD3 and IFI35), were selected for further analysis. Lung Cancer Explorer (LCE) and PanglaoDB were used to determine the gene expression and localization in different cells, and GSVA was used to study the potential biological function of the hub genes. After finding that RR was related to the response module and TME, the Tumor and Immune System Interaction Database (TISIDB) was used to evaluate the correlation between immune cell abundance and the hub genes in the LUAD samples. Finally, we analyzed the expression of the hub genes and the efficacy of drugs derived from the Computational Analysis of Resistance (CARE) database to provide potential complementary therapy for RR patients.

## Materials and Methods

### Data Collection

Transcriptome RNA-seq data of 513 LUAD tumor samples and the corresponding clinical data were downloaded from TCGA (release-18.0) database (https://portal.gdc.cancer.gov/) with level 3. Based on the TCGA sample phenotype, we defined the patients with complete response (CR)/partial response (PR) as RS, and those with progressive disease (PD)/stable disease (SD) as RR.

### Gene Set Variation Analysis

We utilized the GSVA (Hanzelmann et al., 2013) R package version 1.34.0 to find the pathways most associated with RT and the potential function of RT-related hub genes by setting kcdf = “Poisson,” min.sz = 20, max.sz = 500. Fifty samples with RT data in Table 1 were selected for GSVA analysis, and both top 25 terms enriched in RR or RS groups were selected for heatmap display (Kolde, 2019).

**TABLE 1:** Baseline characteristics of patients (N = 126).

	Characteristic	Median (range) or n (%)
Age (y)		64 (40–88)
Sex	Male	64 (50.79)
	Female	62 (49.21)
T stage	T1	25 (19.84)
	T2	81 (64.29)
	T3	15 (11.90)
	T4	4 (3.17)
	TX	1 (0.79)
N stage	N0	59 (46.83)
	N1	33 (26.19)
	N2	30 (23.81)
	N3	2 (1.59)
	NX	2 (1.59)
M stage	M0	87 (69.05)
	M1	8 (6.35)
	MX	31 (24.60)
Chemotherapy response	CR/PR (sensitive)	68 (53.97)
	PD/SD (resistance)	34 (26.98)
Radiotherapy response	CR/PR (sensitive)	31 (24.60)
	PD/SD (resistance)	19 (15.08)

Note: y, years; n, number of patients; T, tumor; N, nodal status; M, metastasis; PD, progressive disease; SD, stable disease; PR, partial response; CR, complete response.

Through GSVA analysis for a single gene, 513 TCGA-LUAD tumor samples were divided into a high expression group and a low expression group based on the median gene expression to reveal the gene potential function. In addition, the “Limma” R package version 3.42.2 (Ritchie et al., 2015) was used to find the different essential pathways of LUAD. Adjusted *p* < 0.001 was regarded as statistically significant. The gene sets of “c2.cp.kegg.v7.0.symbols.gmt,” “c2.cp.reactome.v7.0.symbols.gmt,” “c5.all.v7.0.symbols.gmt,” and “c7.all.v7.0.symbols.gmt” were downloaded from the Molecular Signature Database (MSigDB, http://software.broadinstitute.org/gsea/downloads.jsp) (Liberzon et al., 2015) as the reference.

### Weighted Gene Co-Expression Network Construction

The R package Weighted Correlation Network Analysis (WGCNA) version 1.70-3 (Langfelder and Horvath, 2008) was applied to find clinical trait-related modules and hub genes among them. The adjacency matrix was transformed into a topological overlap matrix (TOM). According to the TOM-based dissimilarity measure, genes were divided into different gene modules. Here, we set the soft-thresholding power to 3 (scale-free *R* ^2^ = 0.85, networkType = “signed hybrid”), the cut off to 0.25, deepSplit to 3, TOMType to unsigned, corType to pearson, and the minimal module size to 50 to identify key modules. The module with the highest and lowest correlation with RT was selected to explore its biological function through ClueGO analyses. Hub genes were defined as those with eigengene connectivity (kME) > 0.8.

### Gene Function Enrichment and Pathway Analysis

We conducted Gene Ontology (GO) enrichment and Kyoto Encyclopedia of Genes and Genomes (KEGG) pathway analysis using Cytoscape version 3.7.2 (Doncheva et al., 2019) software “ClueGO” (Bindea et al., 2009) as well as the R package “clusterProfiler” version 3.14.3 (Yu et al., 2012). GO term or KEGG pathways with adjusted *p* < 0.05 were considered statistically significant and visualized by “ClueGO” and “Enrichplot” (Yu, 2018).

### Analysis of Neoantigen-Coding Genes of RT Samples

The Cancer Immunome Atlas (https://tcia.at/home; Charoentong et al., 2017) is widely used in the query for gene expression of neoantigens and cancer germline antigens, by which we collected the neoantigen-coding genes of RR and RS samples with default parameters. We got the list of neoantigen-coding genes by choosing the “Neoantigens” tab after inputting IDs of RS and RR patients in the TCIA filter. In addition, a web tool for list comparison, jvenn (http://jvenn.toulouse.inra.fr/app/example.html; Bardou et al., 2014), was used to find different neoantigen-coding genes from the RT samples. GO and KEGG analyses were performed to study the biological functions and associated pathways of the differential neoantigen-coding genes.

### Analysis of Gene Expression in Different Cell Types

To investigate the expression of hub genes in LUAD, we applied the online tool Lung Cancer Explorer (http://lce.biohpc.swmed.edu/lungcancer/metagenename.php; Cai et al., 2019), which contains 56 lung cancer datasets from the Sequence Read Archive (SRA) and Gene Expression Omnibus (GEO) databases. From this database, we collected a meta-analysis of hub gene expression. Then we performed meta-analysis on the LUAD dataset with default parameters. In addition, PanglaoDB, a single-cell gene expression resource database (https://PanglaoDB.se/index.html; Franzen et al., 2019), was used to confirm the cell type expressing hub genes.

### Analysis of Gene Expression and Tumor-Infiltrating Immune Cells

To investigate the correlation between the expression of selected hub genes and the abundance of tumor-infiltrating lymphocytes (TILs), we applied the online tool TISIDB (http://cis.hku.hk/TISIDB/index.php; Ru et al., 2019), which reports 988 genes involved in antitumor immunity. Then we determined the relationship between hub gene expression and tumor-infiltrating immune cells by TISIDB analysis with selection of the tabs for lymphocytes, the LUAD dataset and antigen-presenting cells (APCs).

### Hub Gene Computational Analysis of Drug Efficacy

Computational Analysis of Resistance (CARE, http://care.dfci.harvard.edu/) (Jiang et al., 2018) was utilized to evaluate the hub genes associated with drug efficacy. A positive CARE score indicates a higher expression value to be associated with drug response, while a negative score indicates drug resistance. CARE can evaluate the relationship between hub genes and drug therapy and can be used as an alternative therapy for RR. The 3D structures of drugs were downloaded from the PubChem database (http://pubchem.ncbi.nlm.nih.gov/).

### Score Calculation of Tumor-Infiltrating Immune Cells

To verify the differences of tumor-infiltrated immune cells between RR and RS groups, we used CIBERSORT-ABS in an online tool TIMER2.0 (Newman et al., 2015; Li et al., 2016; Sturm et al., 2019) to calculate the scores of tumor-infiltrated immune cells by tissue transcriptional profiles. The average CIBERSORT-ABS scores of both groups are showed in a bar plot.

## Results

### Identification of RT-Related Gene Function and Tumor Immune Environment

**FIGURE 1:**
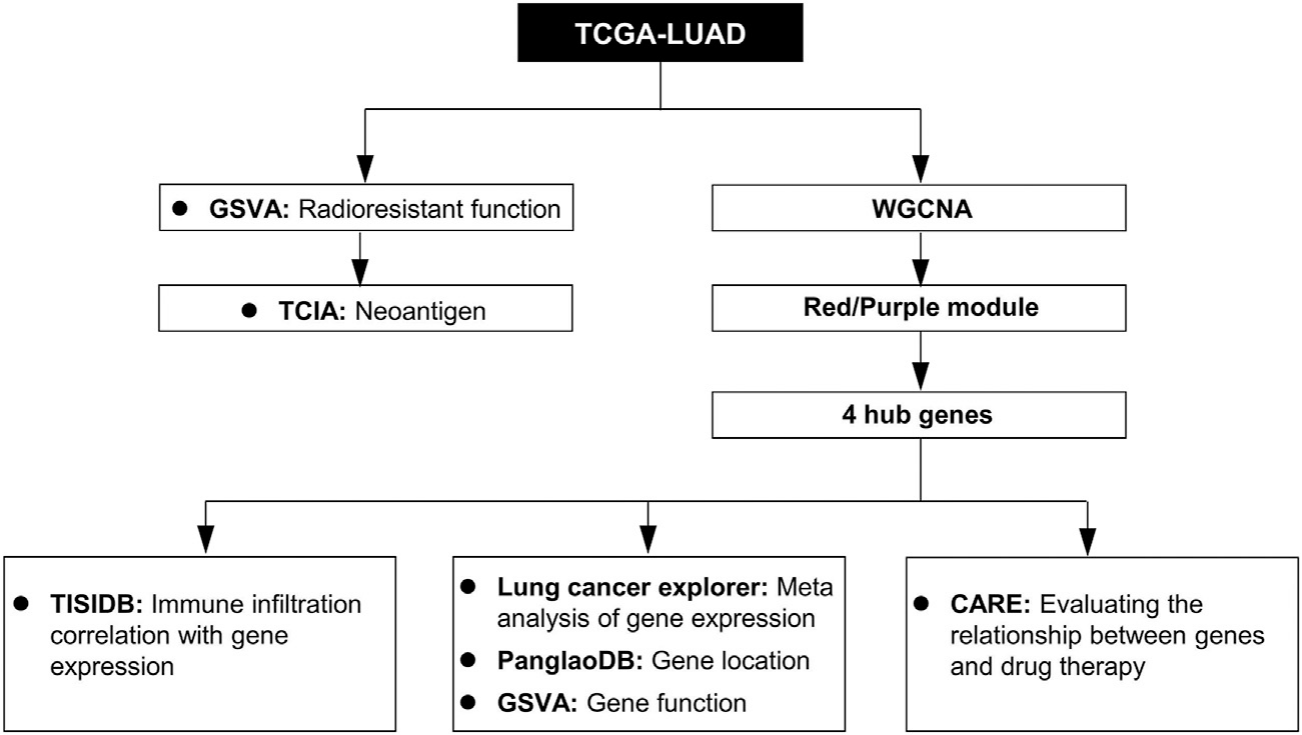
Study workflow. TCGA-LUAD, The Cancer Genome Atlas LUAD datasets; GSVA, gene set variation analysis; WGCNA, weighted gene co-expression network analysis; TCIA, The Cancer Immunome Database; TISIDB, Tumor and Immune System Interaction Database; PanglaoDB, single-cell RNA sequencing experiments from mouse and human database; CARE, Computational Analysis of Resistance.

**FIGURE 2:**
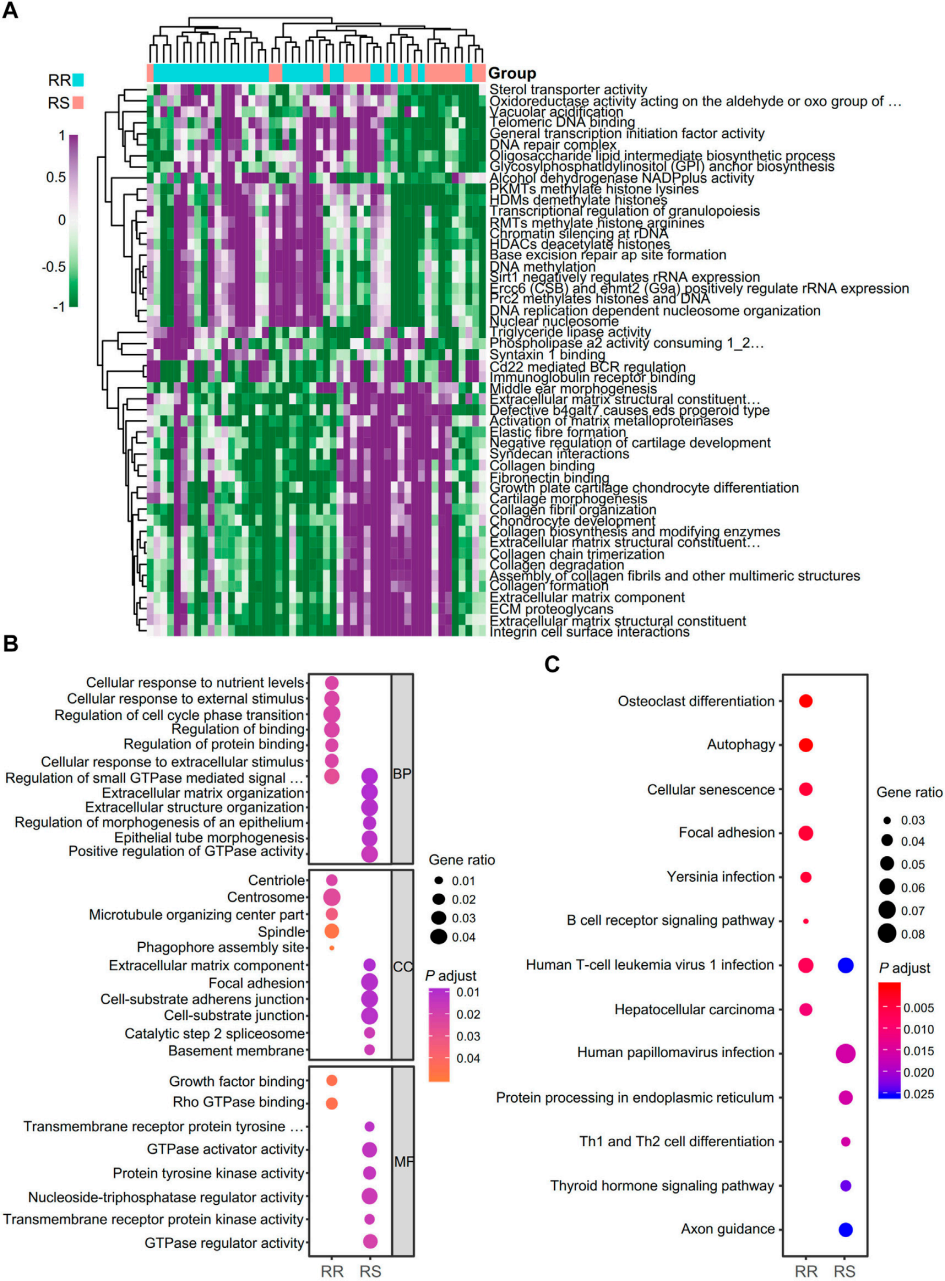
Identification of biological function and neoantigen-coding genes of RT patients. **(A)** Analysis of the biological functions of RT patients with GSVA. RR, radioresistant; RS, radiosensitive. **(B)** GO term analysis for differential neoantigen-coding genes between the RR and RS groups in the TCIA database. **(C)** KEGG analysis of differential neoantigen-coding genes between the RR and RS groups in the TCIA database.

Data analysis flow chart was shown in Figure 1. To find the difference between the RR and RS groups, we utilized GSVA to distinguish the biological function of the RR and RS samples (Figure 2A). Based on GSVA analysis, we found that the functional difference between RS and RR patients was associated with extracellular matrix (ECM) and DNA-related functions, which was consistent with previous reports (Ljungman, 2009; Bian et al., 2020; Haeger et al., 2020). Interestingly, two immune-related terms, immunoglobulin receptor binding and CD22-mediated BCR regulation (Figure 2A), were observed in the list of the top 25 terms enriched in the RR or RS groups, indicating that the immune microenvironment is involved in the RT response.

To identify neoantigen-coding genes in RR and RS patients, the TCIA database was used for analysis, and we found that there were only 13.6% neoantigen-coding genes with overlap. To address the different functions of RS and RR neoantigen-coding genes, GO and KEGG analyses were performed. In GO term analysis, we observed that the RR group was mainly related to the terms of cellular response to nutrient levels, cellular response to external stimulus, regulation of small GTPase mediated signal transduction, regulation of cell cycle phase transition, regulation of binding, centriole, spindle, microtubule organizing center part, growth factor binding and Rho GTPase binding, while the RS group was closely related to the terms extracellular matrix organization, regulation of morphogenesis of an epithelium, epithelial tube morphogenesis, positive regulation of GTPase activity, focal adhesion, cell-substrate junction, catalytic step 2 spliceosome, basement membrane, protein tyrosine kinase activity, GTPase regulator activity and transmembrane receptor protein kinase activity (Figure 2B).

The results of KEGG pathway analysis demonstrated that the RR group was related to the terms osteoclast differentiation, autophagy, cellular senescence, focal adhesion and B cell receptor signaling pathway, while the RS group was mainly related to human papillomavirus infection, protein processing in the endoplasmic reticulum (ER), Th1 and Th2 cell differentiation, thyroid hormone signaling pathway and axon guidance (Figure 2C).

We used CIBERSORT-ABS in TIMER2.0 to identify the tumor-infiltrating immune cell types of RR and RS patients according to a previous study (Yi et al., 2021; Supplementary Figure S1A). The proportion of the immune cells between the RR and RS groups was different, especially the B cells, monocytes, myeloid dendritic cells and eosinophils (Supplementary Figure S1B).

### Validation of RT-Related Gene Modules in the TCGA-LUAD Dataset

**FIGURE 3:**
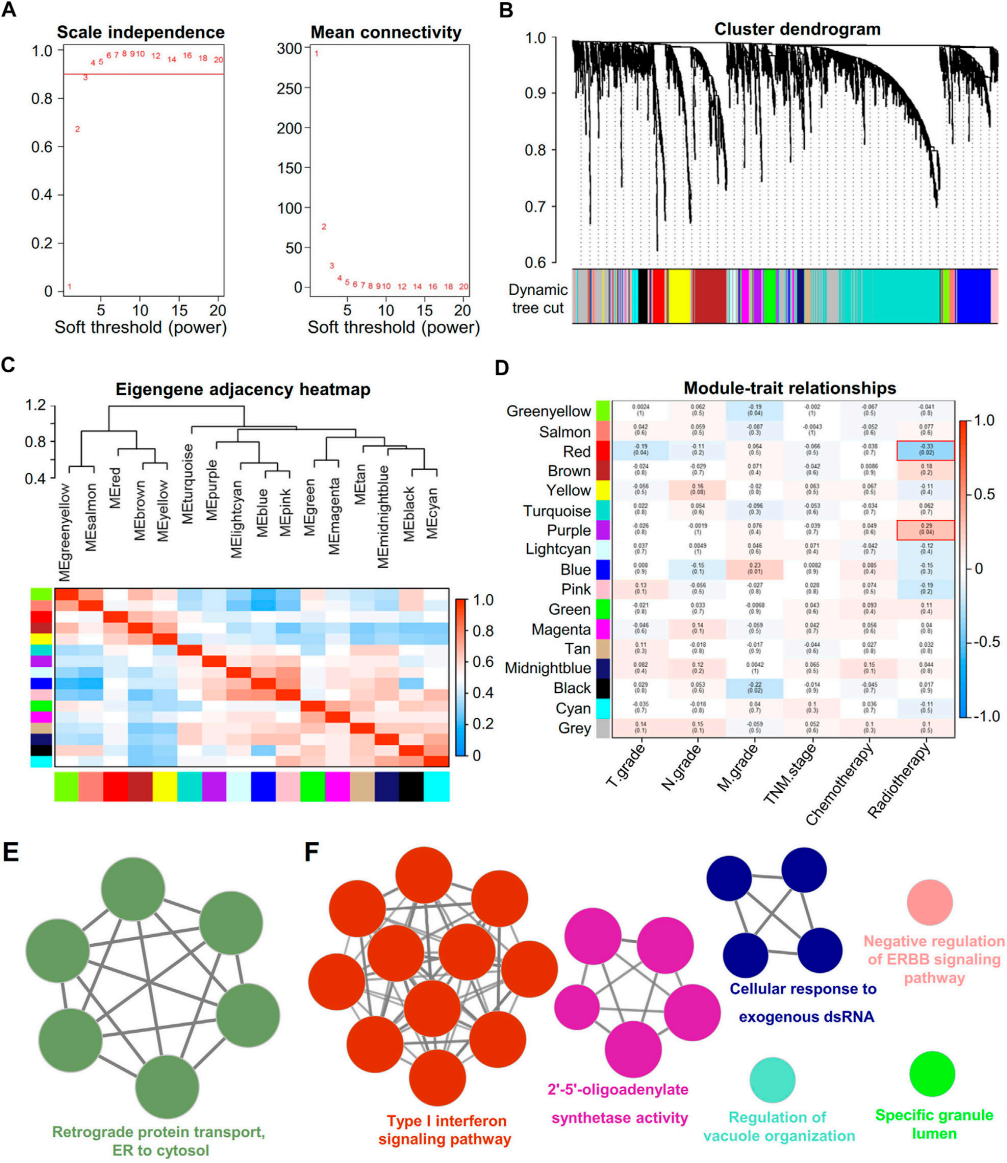
Identification of key modules correlated with clinical traits in the TCGA-LUAD dataset with WGCNA and ClueGO. **(A)** Soft thresholding for WGCNA analysis. **(B)** Clustering dendrogram of genes based on topological overlap. **(C)** Clustering of module eigengenes. The heatmap shows the correlation of the adjacency module. **(D)** Heatmap of the correlation between module eigengenes and clinical traits of RT. **(E)** Enrichment analysis of the red module. Each node represents an enrichment pathway. **(F)** Enrichment analysis of the purple module. Each node represents an enrichment pathway, and the circle color indicates different functional groups.

The data of therapeutic effect on patients who accepted either RT or chemotherapy or a combination of both treatments are shown in Table 1 as the clinical case information. To identify the key modules of RT, we performed WGCNA with the data from 126 patients (Table 1). By setting the soft-thresholding power as 3 (scale-free *R* ^2^ = 0.85) and the cut height as 0.25, we identified 17 modules (Figures 3A–C). From the module-trait correlation heatmap, two modules were identified with close relation to RT. The purple module was significantly correlated with RS, and the red module was significantly correlated with RR; neither module was related to chemotherapy (Figure 3D). ClueGO was used to reveal the potential biological functions and pathways of RR and RS modules. Our results demonstrate that the RS module is related to the type I interferon pathway (Figure 3E), which is in agreement with previous reports (Burnette et al., 2011; Wilkins et al., 2019). In contrast, the RR module displays a close relation with protein assembly transport (Figure 3F).

### Identification of Hub Genes in RR and RS Modules

Genes in the RR and RS modules with kME > 0.8 are displayed in Tables 2, 3, with descending order on kME. Most of the genes in the RR module belong to the immunoglobulin family. Interestingly, some RR module genes, such as PIM2 (Guo et al., 2005) (kME = 0.953), did not belong to the immunoglobulin family but were reported to be associated with RT. Then, we selected the top two genes MZB1 (kME = 0.928) and DERL3 (kME = 0.863) other than immunoglobulin and previously reported genes as the hub genes. In the RS module, we selected hub genes with the highest kME values, including PSMD3 (kME = 0.924) and IFI35 (kME = 0.893), for further analysis.

**TABLE 2:** RR module gene (kME > 0.8) from WGCNA.

Ensemble ID	Gene symbol	kME	Type	Location	Description
ENSG00000102096	PIM2	0.953494	Protein coding	Xp11.23	Pim-2 proto-oncogene, serine/Threonine kinase
ENSG00000211592	IGKC	0.944670	Protein coding	2p11.2	Immunoglobulin kappa constant
ENSG00000170476	MZB1	0.928659	Protein coding	5q31.2	Marginal zone B and B1 Cell specific protein
ENSG00000253755	IGHGP	0.890621	Pseudogene	14q32.33	Immunoglobulin heavy constant gamma P
ENSG00000211677	IGLC2	0.884386	Protein coding	22q11.22	Immunoglobulin lambda constant 2
ENSG00000211896	IGHG1	0.883868	Protein coding	14q32.33	Immunoglobulin heavy constant gamma 1
ENSG00000099958	DERL3	0.863413	Protein coding	22q11.23	Derlin 3
ENSG00000211648	IGLV1-47	0.840419	Protein coding	22q11.22	Immunoglobulin lambda variable 1–47
ENSG00000211945	IGHV1-18	0.838534	Protein coding	14q32.33	Immunoglobulin heavy variable 1–18
ENSG00000224373	IGHV4-59	0.833868	Protein coding	14q32.33	Immunoglobulin heavy variable 4–59
ENSG00000239819	IGKV1D-8	0.829594	Protein coding	2p11.2	Immunoglobulin kappa variable 1D-8
ENSG00000211593	IGKJ5	0.823355	Protein coding	2p11.2	Immunoglobulin kappa joining 5
ENSG00000231292	IGKV1OR2-108	0.820230	Protein coding	2q14.1	Immunoglobulin kappa variable 1/OR2-108
ENSG00000211897	IGHG3	0.813846	Protein coding	14q32.33	Immunoglobulin heavy constant gamma 3
ENSG00000270550	IGHV3-30	0.812538	Protein coding	14q32.33	Immunoglobulin heavy variable 3–30
ENSG00000211956	IGHV4-34	0.809769	Protein coding	14q32.33	Immunoglobulin heavy variable 4–34
ENSG00000211966	IGHV5-51	0.805408	Protein coding	14q32.33	Immunoglobulin heavy variable 5–51
ENSG00000211679	IGLC3	0.803635	Protein coding	22q11.22	Immunoglobulin lambda constant 3
ENSG00000211965	IGHV3-49	0.803072	Protein coding	14q32.33	Immunoglobulin heavy variable 3–49

kME: eigengene-based connectivity. Type: gene type. Location: chromosome localization information.

**TABLE 3:** RS module gene (kME > 0.8) from WGCNA.

Ensemble ID	Gene symbol	kME	Type	Location	Description
ENSG00000108344	PSMD3	0.924858	Protein coding	17q21.1	Proteasome 26S subunit, non-ATPase 3
ENSG00000068079	IFI35	0.893972	Protein coding	17q21.31	Interferon induced protein 35
ENSG00000141696	P3H4	0.893253	Protein coding	17q21.2	Prolyl 3-hydroxylase family member 4
ENSG00000141698	NT5C3B	0.877200	Protein coding	17q21.2	5′-Nucleotidase, cytosolic IIIB
ENSG00000131475	VPS25	0.868741	Protein coding	17q21.2	Vacuolar protein sorting 25 homolog
ENSG00000277791	PSMB3	0.868408	Protein coding	17q12	Proteasome subunit beta 3
ENSG00000108774	RAB5C	0.849465	Protein coding	17q21.2	RAB5C, member RAS oncogene family
ENSG00000002834	LASP1	0.844632	Protein coding	17q12	LIM and SH3 protein 1
ENSG00000278845	MRPL45	0.829890	Protein coding	17q12	Mitochondrial ribosomal protein L45
ENSG00000136448	NMT1	0.822686	Protein coding	17q21.31	N-Myristoyltransferase 1
ENSG00000141756	FKBP10	0.822572	Protein coding	17q21.2	FKBP prolyl isomerase 10
ENSG00000131462	TUBG1	0.807538	Protein coding	17q21.31	Tubulin gamma 1
ENSG00000137563	GGH	0.803600	Protein coding	8q12.3	Gamma-glutamyl hydrolase

kME: eigengene-based connectivity. Type: gene type. Location: chromosome localization information.

### Hub Gene Expression in Different Cells in Lung Adenocarcinoma

**FIGURE 4:**
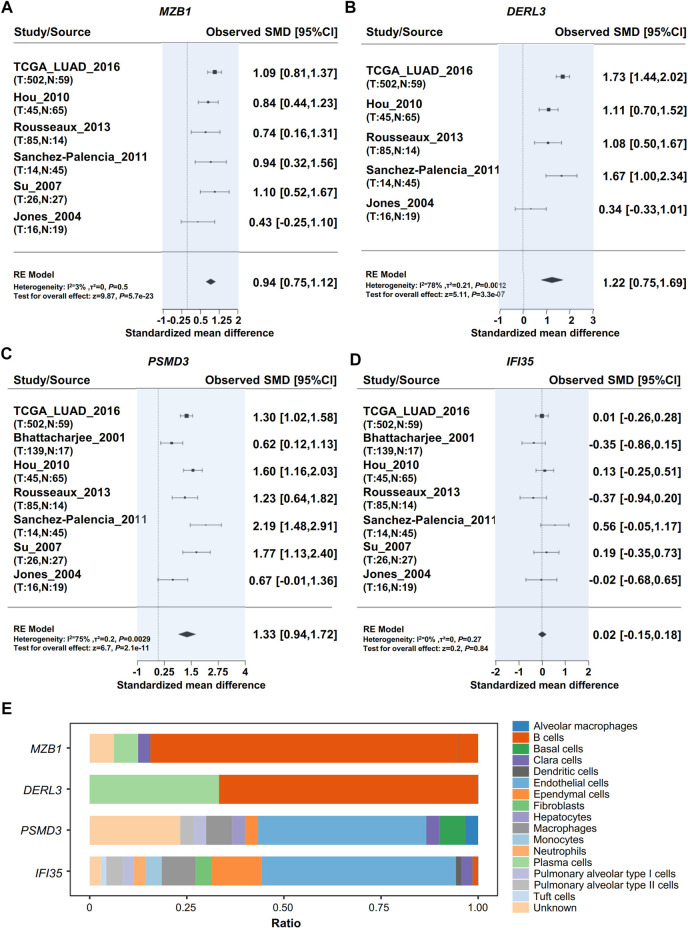
Validation of hub gene expression. Meta-analysis of Hub gene expression by using the Lung Cancer Explorer database. **(A)** MZB1, **(B)** DERL3, **(C)** PSMD3, **(D)** IFI35. **(E)** Analysis of cells expressing RR hub genes (MZB1, DERL3) and RS hub genes (PSMD3, IFI35) in the lung tissue with PanglaoDB.

We performed a meta-analysis of those four genes in different datasets from the Lung Cancer Explorer, and the results are shown in Figures 4A–D. It was noted that three of the four genes, i.e., MZB1, DERL3, and PSMD3, were more highly expressed in cancer tissues than in adjacent normal lung tissues (Figures 4A–D). Then, we used PanglaoDB to explore gene expression in different cells and found that the RR module genes MZB1 and DERL3 were mainly expressed in B cells and plasma cells, while IFI35 and PSMD3 were mainly expressed in endothelial cells (Figure 4E). The results hint that the RR genes are expressed in B cells infiltrated into the TME, and the RS genes are mainly expressed in tumor tissues.

### GSVA Reveals a Close Relationship Between Hub Genes and the Immune Microenvironment

**FIGURE 5:**
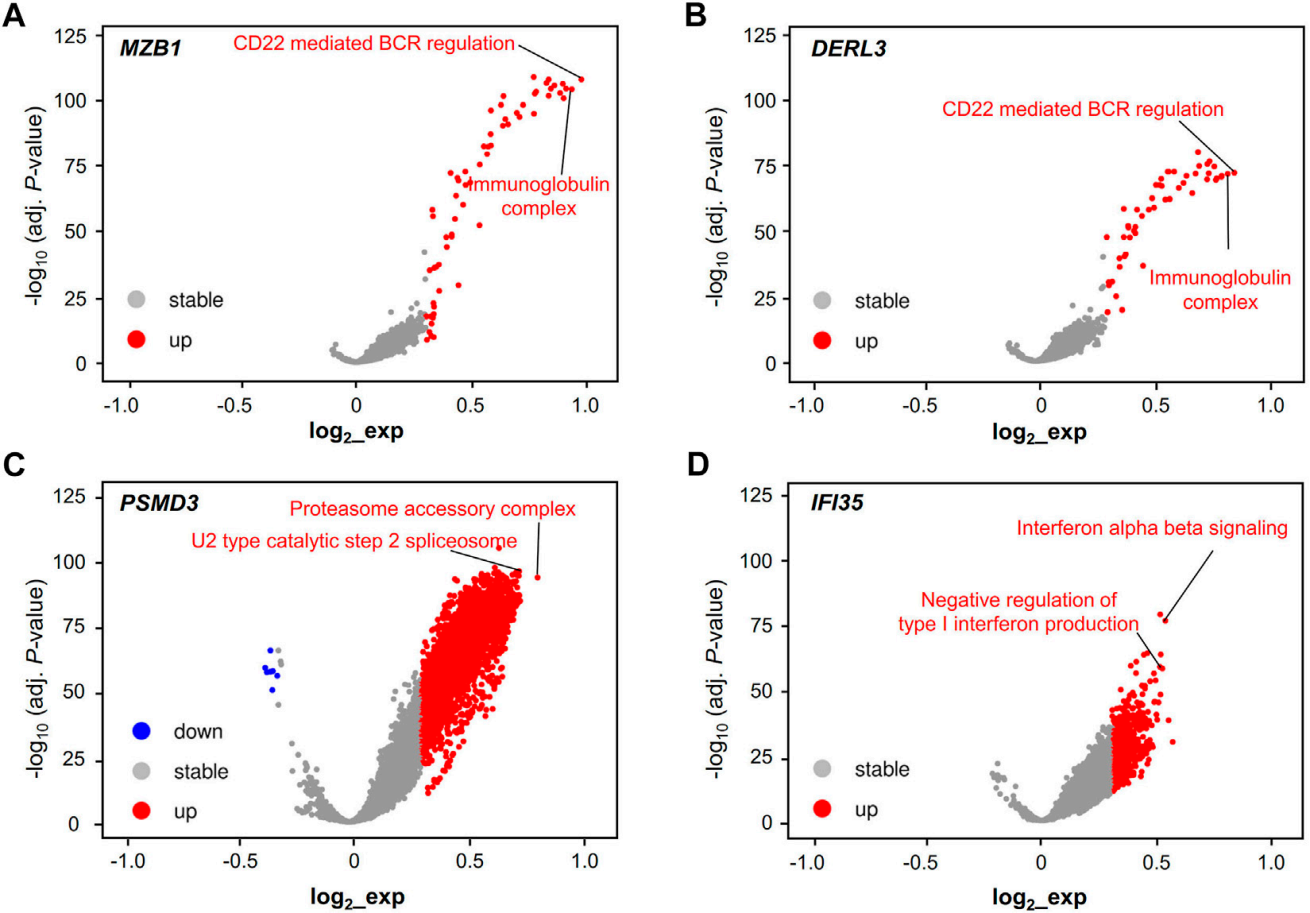
Validation of the cell type and function of hub genes. Functional volcano plot of RR hub genes MZB1 **(A)** and DERL3 **(B)** and RS hub genes PSMD3 **(C)** and IFI35 **(D)** in the TCGA-LUAD dataset. Gene set variation analysis (GSVA) and the “Limma” *R* package were used to find the different essential pathways of LUAD for the single hub genes, including MZB1, DERL3, PSMD3, and IFI35. Adjusted *p* < 0.001 and log_2_ (fold change) > |0.3| were regarded as statistically significant.

We performed GSVA to further study the potential function of these RR and RS genes in LUAD. The potential functions of the RR genes MZB1 and DERL3 were significantly enriched in terms of CD22-mediated BCR regulation and immunoglobulin receptor binding (Figures 5A,B), while the RS genes PSMD3 and IFI35 displayed a different functional category. PSMD3 shows a significant association with the proteasome accessory complex and U2 type catalytic step 2 spliceosome, and IFI35 is mainly related to the function of interferon α–β signaling and the negative regulation of type I interferon production (Figures 5C,D).

### Correlation Between RT Hub Gene Expression and Antigen-Presenting Cells in Lung Adenocarcinoma

**FIGURE 6:**
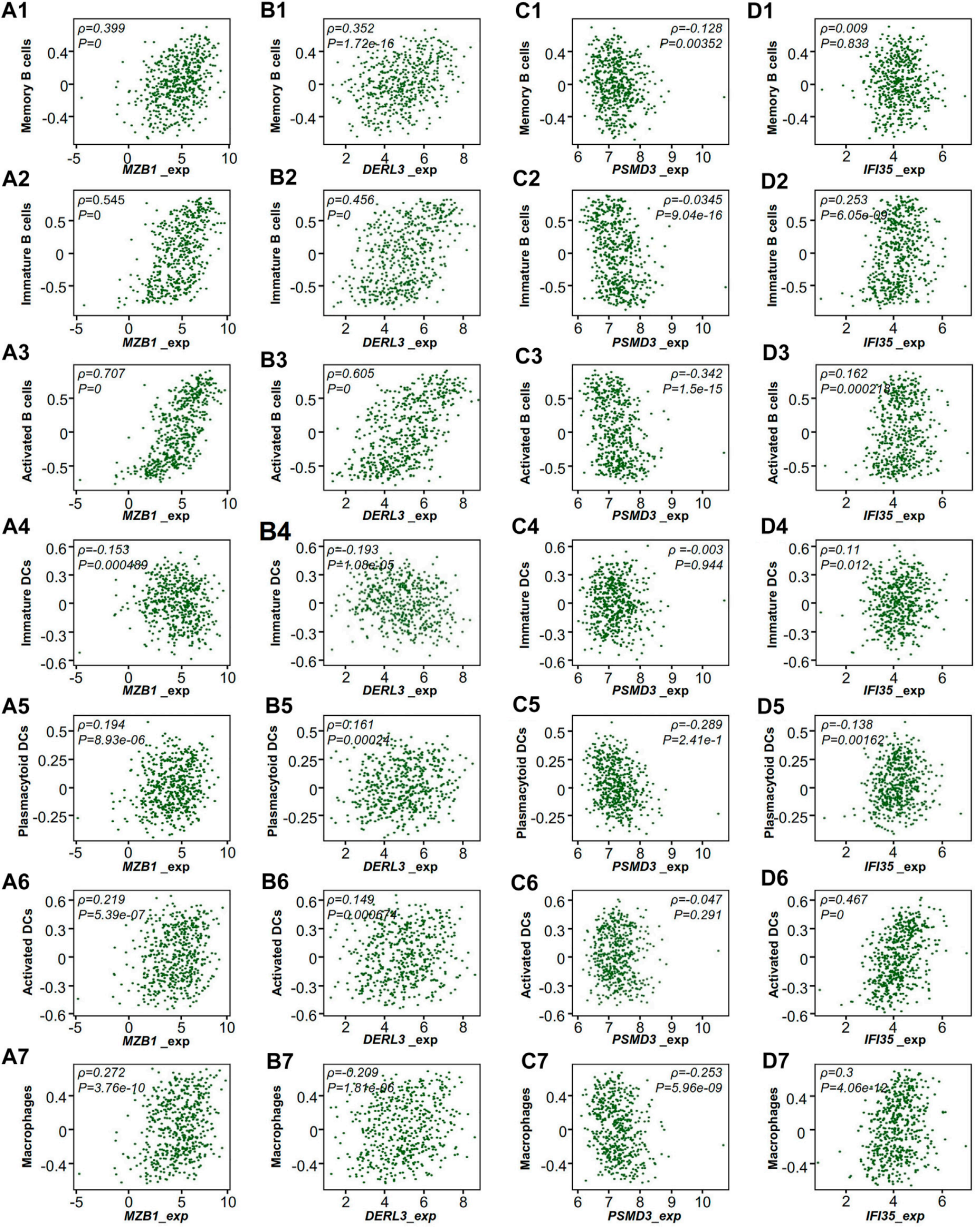
TCGA-LUAD microenvironment of RT-treated patients. Correlation of the expression of hub genes with the infiltration of immune cells from TISIDB. **(A)** MZB1, **(B)** DERL3, **(C)** PSMD3, **(D)** IFI35. Each dot represents a sample in the TCGA-LUAD dataset. *ρ*, Spearman correlation coefficient.

Tumors were engulfed in a complex microenvironment, which critically impacts disease progression and response to therapy (Quail and Joyce, 2013). We used TISIDB to explore the relationship between the expression of hub genes and the infiltration of APCs (Slatter et al., 2016). Interestingly, we found that the infiltration of APCs, as well as B cells, had a positive correlation with RR but not RS hub genes (Figure 6).

### Prediction of Potential Therapy for RR Treatment

**FIGURE 7:**
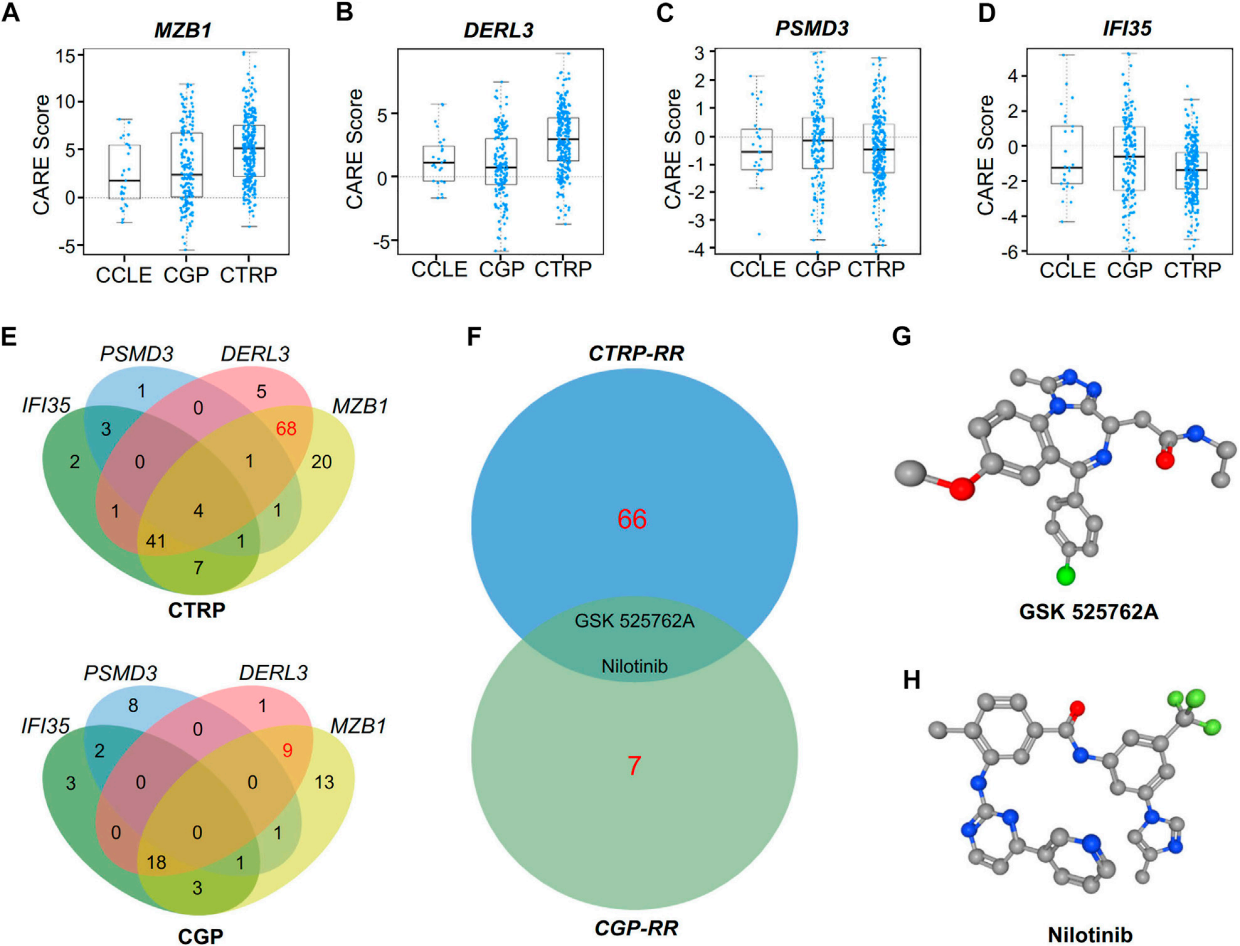
CARE analysis for hub genes in response to targeted therapy. **(A–D)** CARE analysis of the resistance module genes *MZB1* **(A)** and *DERL3* **(B)** and the response module genes *PSMD3* **(C)** and *IFI35* **(D)** in the databases of the Broad Institute Cancer Cell Line Encyclopedia, the CTRP and the Genomics of Drug Sensitivity in Cancer (CGP). **(E)** Venn diagram showing the overlap of target drugs affected by *MZB1*, *DERL3*, *PSMD3*, and *IFI35* in the CTRP and CGP databases. **(F)** Venn diagram showing the potential target drugs for RR patients, i.e., GSK525762A and nilotinib. **(G–H)** Three-dimensional (3D) structures of GSK525762A **(G)** and nilotinib **(H)**.

To study alternative therapy for RR patients, we used the CARE database to analyze the expression of the hub genes as well as the efficacy of drugs. Our results show that the RR hub gene expression in the databases the Broad Institute Cancer Cell Line Encyclopedia (CCLE), the Cancer Therapeutics Response Portal (CTRP) and the Genomics of Drug Sensitivity in Cancer (GDSC, also known as CGP) had a positive correlation with drug efficacy (Figures 7A,B), while the RS genes were associated with drug resistance (Figures 7C,D). Moreover, intersection was performed with the drugs positively correlated with RR genes or negatively correlated with RS genes in the CTRP and CGP databases (Figure 7E).

After exclusion of drugs targeting the RS genes, the drugs targeting both RR genes DERL3 and MZB1 in the CTRP and CGP databases were chosen for intersection analysis to find shared drugs in both databases (Figure 7F). Finally, we obtained the BRD4 inhibitor GSK525762A and the monoclonal antibody nilotinib as potential drugs for RR treatment. The three-dimensional structures of these two drugs are shown in Figures 7G,H.

## Discussion

As one of mainstream cancer therapies, RT has a good effect in a variety of tumors (De Ruysscher et al., 2019; Sia et al., 2020). However, it is not so effective for several cancers, including LUAD (Li et al., 2015). In the clinic, there are many NSCLC patients who are failed to achieve a cure after surgery. In fact, 30–55% of patients with NSCLC develop recurrence and die of their disease despite curative resection (Uramoto and Tanaka, 2014). In most cases, surgery for the patients with NSCLC does not mean complete removal of cancer cells, especially for the patients received RT. RT is used as a post-surgical adjuvant therapy selectively for the patients at a risk of incomplete removal of cancer cells after surgery. Recent studies demonstrate that subsequent immune responses have a significant impact on the efficacy of RT, except for direct damage to cancer cells caused by high-energy rays (Dovedi et al., 2016; Rodriguez-Ruiz et al., 2020).

Although microarray and RNA-seq techniques have been applied in the study of LUAD, the mechanism by which RT affects the TME is still not well understood. The classical radiobiology dogma fails to consider the effect of RT on TME, however, the response of TME to RT might be significant for the success of treatment (Dai et al., 2020). To explore the factors affecting RT, we utilized GSVA to distinguish the biological functions of RR and RS tumors, indicating that the TME was involved in the RT response. Based on the association between the RT effect and the TME, we evaluated the different tumor neoantigens from RR and RS patients by GO and KEGG analysis and found that the TME significantly affected RT efficacy.

In the present study, we performed bioinformatic analysis of the gene transcriptional profiles in LUAD patients subjected to RT, which demonstrated that B cells in TME were associated with RR. B cells are the main humoral immune cells derived from hematopoietic stem cells that play an essential role in antitumor immunity. Apart from positive regulation of antitumor immune process, tumor-infiltrating B cells (TIB) can also negatively regulate antitumor immune response (Guo and Cui, 2019). In this study, we also used WGCNA to explore new hub genes associated with RT and found that the RR module was mainly composed of immunoglobulin-related genes. ClueGO analysis results demonstrated that ER to cytosol retrograde protein transport was enriched in the RR module, indicating that the RR module is involved in the term ER to cytosol retrograde protein transport. The association of TIB with RR may be related to the dysfunction of immunoglobulin generated process by B cells. In contrast, the biological function of the RS module was closely related to the type I interferon pathway, which has been reported to play an important role in the immune response of RT (Burnette et al., 2011; Feng et al., 2020). Therefore, further studies on the roles of hub genes in RR and RS modules are needed.

Several studies had reported inconsistent roles for RR hub genes, such as MZB1 and DERL3, in various malignancies. In hepatocellular carcinoma, patients with high MZB1 expression were found with a better prognosis. To the contrast of hepatocellular carcinoma, high MZB1 expression was associated with poor prognosis of patients with breast cancer. Interestingly, DERL3 expression was found to be positively correlated with MZB1 expression in the clinical specimens from breast cancer patients, suggesting DERL3 may also participate tumor progression (Watanabe et al., 2020). GSVA analysis results demonstrated that the RR genes are related to the immunoglobulin complex, indicating that they are closely related to B cells.

As for RS hub genes, high PSMD3 expression was associated with poor prognosis in the breast cancer patients (Fararjeh et al., 2019). In addition, IFI35 was reported to be involved in the regulation of radiosensitivity of colorectal cancer (CRC) cells, indicating that IFI35 might be a RT target for CRC (Hu et al., 2021). Intriguingly, the RS hub genes PSMD3 and IFI35 were found in the same GO term “positive regulation of defense response” (Subramanian et al., 2005; Watanabe et al., 2020), indicating that they are closely related to reactivity of RT.

Importantly, the GSVA result showed that the RS representative genes PSMD3 and IFI35 were associated with cellular homeostasis and immune responses, including the proteasome accessory complex and interferon α–β signaling, reflecting the characterization of tumors and immune responses induced by RT. Interestingly, we found that RS genes are mainly located in genomes 17q21 and 17q12, which indicates that the short arm of chromosome 17 contains some essential genes that influence the RT effect on LUAD.

To study alternative therapy for RR patients, we selected the efficacy of specific gene-associated drugs. We found that two RR gene-associated drugs, i.e., nilotinib and GSK525762A, were shared by CTRP and CGP databases. Intriguingly, nilotinib (Sun et al., 2019) and GSK525762A (Klingbeil et al., 2016; Zhang et al., 2018) have been reported to inhibit LUAD cell proliferation or growth. Our study provides drug candidates for the therapy of LUAD patients with RR.

In general, by integrating GSVA, WGCNA and other immune microenvironment databases, we found that the RR of LUAD might be associated with the infiltration of immune cells, especially B cells, which was in line with the finding that B cells are a key factor promoting carcinogenesis by immunosuppression (Schioppa et al., 2011; Klinker and Lundy, 2012). It is reasonable to conclude that the composition of the TME may have a significant impact on the treatment of LUAD. Furthermore, our novel findings on the expression of the hub genes and the efficacy of specific gene-associated drugs provide potential drug candidates for RR patients. However, more studies are required to reveal the effectiveness of the drugs in the treatment of LUAD.

## Data Availability

The original contributions presented in the study are included in the article/Supplementary Material, further inquiries can be directed to the corresponding authors.
