# Supplementary material for: Identification of Radiotherapy-Associated Genes in Lung Adenocarcinoma by an Integrated Bioinformatics Analysis Approach
Source: Front Mol Biosci. 2021 Jun 15;8:624575. doi: 10.3389/fmolb.2021.624575 (PMC8239180; doi:10.3389/fmolb.2021.624575)
Supplement: Supplementary file 1 [file Image1.pdf]

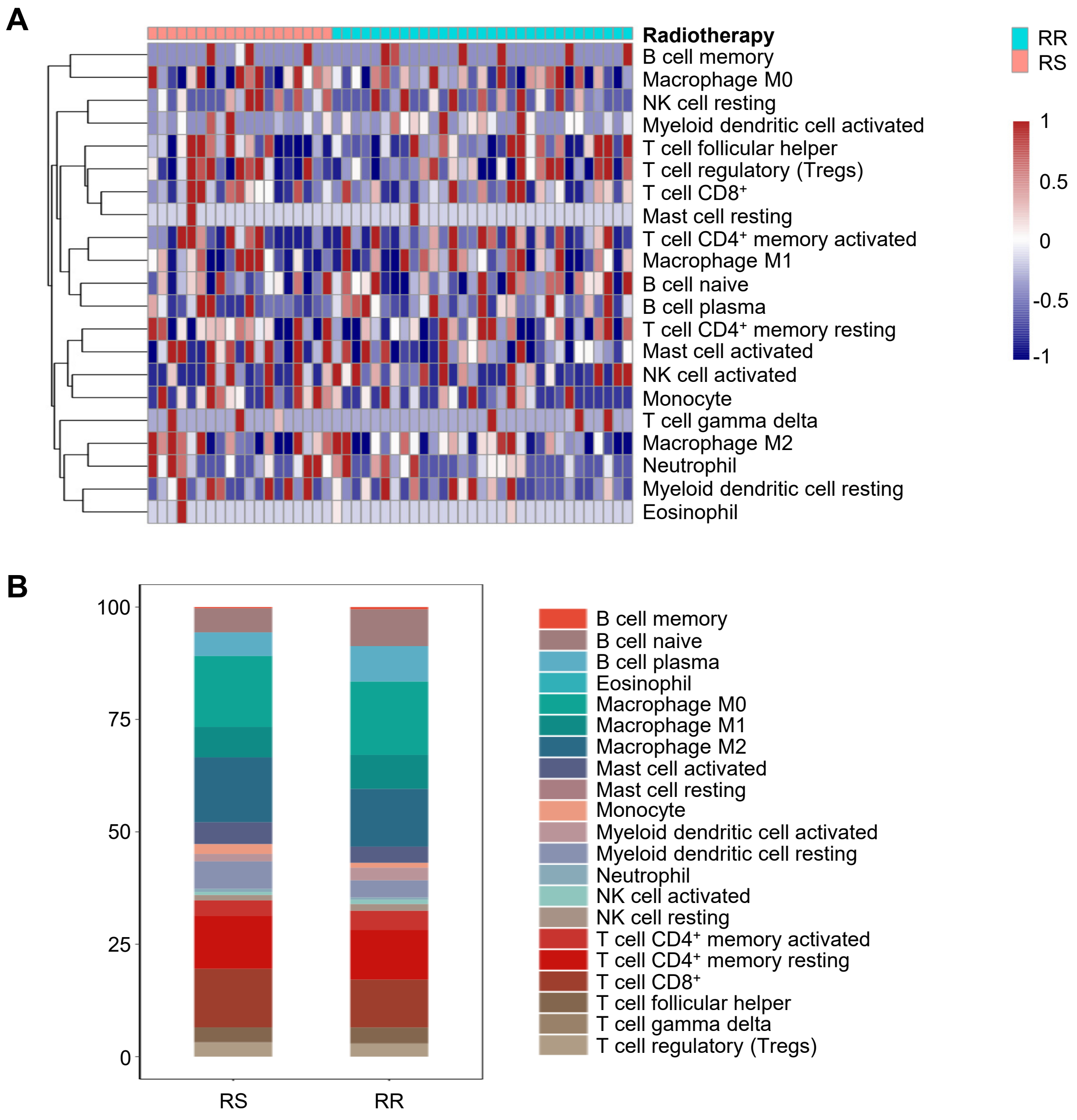

Supplementary Figure 1. Tumor-infiltrating immune cells of RR and RS samples from LUAD patients using CIBERSORT-ABS in TIMER2.0. (**A**) The heatmap shows the CIBERSORT-ABS scores of tumor-infiltrating immune cells in RR and RS groups. (**B**) The barplot shows the proportion of tumor-infiltrating immune cells in the RR and RS groups.
